# Toward a dual-pathway model of neuroplastic adaptation in sport: neural efficiency and neural optimization

**DOI:** 10.3389/fnins.2026.1777591

**Published:** 2026-04-14

**Authors:** Murad Sultanov, Tukezban Rustamova, Aytan Huseynova, Gulchin Mammadova, Khadidja Ismailova

**Affiliations:** 1 Institute of Physiology Named After Academician A. Garayev, Ministry of Science and Education, Republic of Azerbaijan, Baku, Azerbaijan; 2 Department of Anatomy, Physiology and Zoology, Ganja State University, Ganja, Azerbaijan; 3 Department of Hygiene and Food Safety, Azerbaijan State Agricultural University, Ganja, Azerbaijan

**Keywords:** EEG, fMRI, fNIRS, football, frontal lobe, parietal lobe, soccer

## 1 Introduction

In recent years, the number of studies investigating the neural efficiency hypothesis (NEH) in athletes has increased substantially. Soccer, one of the most popular sports worldwide, has also attracted growing attention due to the complex cognitive and sensorimotor demands associated with continuous training and competition. A systematic review of neural efficiency in sports has revealed a heterogeneous pattern of findings: while some studies report decreased activation in experts, others yield inconsistent or even paradoxical results, depending on task characteristics and measurement modality (Li and Smith, 2021).

Many classical demonstrations of neural efficiency originate from stable laboratory paradigms in which stimulus–response mappings are highly predictable. In contrast, soccer represents a prototypical open-skill domain characterized by uncertainty, time pressure, and continuous interaction with dynamically changing stimuli (Heilmann et al., 2022; Sultanov, 2024). Under such conditions, optimal performance may require sustained vigilance and rapid reallocation of attentional resources rather than a global reduction of neural activity. Therefore, direct transfer of predictions derived from closed tasks to complex team sports may be problematic.

To date, empirical findings in soccer remain mixed. Several studies reported results interpreted as supporting neural efficiency in soccer players (Huang et al., 2023; Schmaderer et al., 2023), whereas other investigations have found contrasting evidence (Bishop et al., 2013; Del Percio et al., 2019; Sultanov and Ismailova, 2020; Wright et al., 2013). However, a recent study using a controlled motor–cognitive dual-task paradigm also reported reduced prefrontal activation in soccer players compared to controls (Yin et al., 2025). Additionally, national-level soccer players exhibited lower activation in the left and right dorsolateral prefrontal cortex, the frontal pole area, and the right ventrolateral prefrontal cortex compared to amateurs (Yin et al., 2024).

This review aims to investigate whether a domain-specific reinterpretation of the NEH is necessary in the context of soccer. We propose that in high-uncertainty environments like soccer, the concept of “neural efficiency” should evolve from a global reduction in cortical metabolic cost to neural optimization—a state characterized by the strategic recruitment of specific executive networks and enhanced functional connectivity, rather than universal hypoactivation. This perspective implies that the neural signature of expertise is shaped by the ecological demands of the sporting discipline.

## 2 Review of recent soccer studies

### 2.1 Studies reporting neural efficiency in soccer players

Several recent neuroimaging studies have reported patterns consistent with neural efficiency in soccer players (Table 1). Notably, many of these investigations employed structured laboratory paradigms, including button-trigger responses, *N*-back tasks, observation of video clips, or simplified soccer-related movements and mental rotation. Within such controlled contexts, findings may partly reflect efficiency under relatively predictable task constraints.

**Table 1:** The reviewed soccer studies show heterogeneous results, with several investigations supporting the NEH, while others report increased activation patterns consistent with neural optimization (*N*=10).

References	Modality	Study task	Expertise level	Key findings	Support for NEH?
Bishop et al. (2013)	fMRI	Anticipation task (video-based prediction of opponent's movements)	High-skill vs. low-skill players	Higher accuracy in experts was associated with greater activation in executive function and oculomotor control regions	No (activation)
Del Percio et al. (2019)	EEG	Visuospatial processing of soccer videos	Football players vs. non-players	Experts showed significantly greater parietal alpha desynchronization (higher activation) during soccer-specific tasks	No (contradicts)
Huang et al. (2023)	fMRI	The motor video observation and button-decision-making tasks	Professional college football players vs. novice football players	The left superior temporal gyrus, left inferior temporal gyrus, and left middle occipital gyrus exhibited greater activation in the novice players	Yes (partial—with activation in the frontoparietal cognitive area)
Iwadate et al. (2005)	EEG (ERP)	Somatosensory stimulation (median and tibial nerves)	Soccer players vs. non-athletes	The N140 amplitudes were larger during upper- and lower-limb tasks and the P300 amplitude and latency were larger and shorter during the lower-limb task compared with non-athletes	No (supports cortical excitation)
Murakami et al. (2008)	EEG (HFO)	Somatosensory stimulation (tibial and median nerves)	Football players vs. non-athletes	The P37-N45 amplitude in football players was significantly larger than those in non-athletes. The number of negative peaks of HFOs from the posterior tibial nerve in football players was significantly larger than those in non-athletes	No (supports cortical excitation)
Schmaderer et al. (2023)	fNIRS	General vs. sport-specific cognitive tasks	Semi-professional soccer players	Lower PFC activity during sport-specific (familiar) tasks compared to general (novel) tasks	Yes (supports for familiar stimuli)
Sultanov and Ismailova (2020)	EEG (mobile)	On-field complex visuomotor and visuospatial tasks	Elite youth soccer players	High engagement of the left FPC during dynamic training compared to rest	No (transient hypofrontality)
Wright et al. (2013)	fMRI	Deception vs. direction identification (point-light stimuli)	Higher-skilled vs. lower-skilled soccer players (males and females)	Experts showed significantly greater activation in the action observation network and executive areas specifically during deception identification	No (activation)
Yin et al. (2024)	fNIRS	Mental rotation (spatial perception)	National-level soccer players vs. physical education college students from football clubs	Lower activation in DLPFC/VLPFC/Frontal pole, but stronger brain network connectivity in experts	Yes (partial)
Yin et al. (2025)	fNIRS	Motor-cognitive tasks (single, *N*-back, dual task)	Football players vs. non-footballers	Lower activation in DLPFC/PMC/SMA but higher interhemispheric and sensory-motor functional connectivity in footballers	Yes (partial)

Studies were classified as supporting NEH when expert athletes demonstrated reduced neural activation relative to controls during task performance. Studies were classified as partially supporting NEH when reduced activation co-occurred with additional mechanisms such as increased functional connectivity or network recruitment. Studies were classified as contradicting NEH when expert athletes showed greater neural activation than controls.

Although elements consistent with neural economy were observed—particularly reduced activation in selected frontal regions—the dominant pattern in some studies involved enhanced functional connectivity and stronger integration between motor and cognitive networks during dual-task performance. These findings extend beyond mere reductions in activation magnitude and instead suggest coordinated, adaptive network organization.

Importantly, laboratory paradigms, even when incorporating motor-cognitive components relevant to soccer, do not fully reproduce the perceptual uncertainty, opponent interaction, and rapid contextual fluctuations characteristic of real match situations. Consequently, observed neural efficiency may preferentially reflect optimized processing within structured and predictable environments. Whether similar dynamics would emerge under ecologically valid, high-uncertainty conditions remains an open question. Under such conditions, expertise may involve additional layers of dynamic adaptation that exceed simple reductions in cortical activation.

### 2.2 Studies not reporting neural efficiency in soccer players

In contrast, other studies have reported increased cortical activation in soccer players relative to controls (Table 1). For instance, Del Percio et al. (2019) observed greater alpha rhythm desynchronization in amateur soccer players within parietal regions during visuospatial processing of soccer video clips. At first glance, such findings appear inconsistent with the neural efficiency hypothesis.

One possible explanation lies in the functional specialization of attentional systems. Neural efficiency has most consistently been reported within frontal regions associated with executive control and endogenous (top-down) attention. In contrast, parietal regions are strongly implicated in stimulus-driven, exogenous attentional processes (Petersen and Posner, 2012; Posner and Petersen, 1990). Soccer, as a dynamic and unpredictable open-skill sport, requires constant reorienting to externally driven cues such as opponent movement and ball trajectory.

From this perspective, increased parietal recruitment may not indicate inefficiency, but rather heightened responsiveness of the ventral attention network under conditions of perceptual uncertainty. In highly dynamic environments, full automatization of parietal processing may be limited, as players cannot fully predict which stimulus will occur next. Thus, elevated parietal activation may reflect processing precision and rapid reorienting rather than excessive neural expenditure.

An additional factor concerns the level of expertise represented in many neuroimaging studies. Participants classified as “experts” often include trained or semi-professional athletes rather than elite, world-class performers. Future studies should explicitly distinguish between collegiate, semi-professional, and elite athletes when interpreting neural signatures of expertise.

It is conceivable that long-term elite training fosters the consolidation of tactical patterns into structured “chunks” (Gobet et al., 2001), enabling more efficient delegation of spatial processing across frontal and parietal networks. In less experienced players, the absence of such consolidated representations may necessitate more stimulus-by-stimulus processing, thereby altering activation patterns. Crucially, comparable activation magnitudes across groups do not necessarily imply identical neural strategies. Similar signal levels may arise from qualitatively different mechanisms—such as temporally precise and selectively tuned processing in experts vs. less efficient or less anticipatory engagement in novices. Thus, equal activation does not equate to equal neural organization. Hence, inter-individual variability in neural strategies may further explain why similar activation magnitudes are observed across athletes with different cognitive styles and expertise levels.

Overall, across the reviewed soccer studies, neural efficiency appears most consistently within frontal regions and under structured task conditions. In contrast, increased parietal recruitment during perceptually dynamic tasks may reflect stimulus-driven attentional demands characteristic of open-skill environments. These observations suggest that neural efficiency may be most detectable during simplified laboratory paradigms, whereas performance in ecologically valid soccer contexts likely engages optimized, dynamically coordinated network activity rather than uniform reductions in cortical activation. This interpretation aligns with evidence indicating that neural efficiency tends to emerge under conditions of low to moderate task difficulty and is most robustly observed in frontal regions (Neubauer and Fink, 2009). However, it must be acknowledged that there is currently a lack of empirical neuroimaging studies in soccer that directly measure attention modes using specific behavioral paradigms. Consequently, our interpretation remains a theoretically grounded proposition rather than a definitive conclusion. We draw this hypothesis from established neurobiological models of attention, such as the dual-network framework, which links parietal activation to exogenous and endogenous shift mechanisms. Future research is needed to empirically map soccer expertise to specific dorsal and ventral attentional dynamics under ecologically valid conditions.

## 3 Discussion

Recent systematic evidence points toward a divergence in neuroplastic adaptations across sport types. While closed-skill disciplines are often associated with patterns consistent with neural economy, open-skill sports tend to demonstrate increased brain volume, enhanced resting-state functional connectivity within executive and somatosensory networks, and stronger task-related recruitment of frontal and parietal regions. In addition, the meta-analytic evidence supporting reduced cortical activation in athletes may reflect the structured and laboratory-based nature of the decision-making paradigms employed. Such conditions resemble closed-skill environments, where stimulus-response mappings are stable and internal models can be efficiently automated (Du et al., 2022). In contrast, real-world open-skill sports such as soccer require continuous adaptation to unpredictable contextual changes, potentially favoring neural optimization over global hypoactivation.

### 3.1 NEH in closed-skill sports

In closed-skill sports, long-term training leads to stable structural and functional adaptations consistent with classical efficiency interpretations, as seen in ice-skating athletes (Zhang et al., 2021). Moreover, a systematic review among swimmers demonstrated clear evidence supporting the NEH in this closed-skill activity (Gkintoni et al., 2026). Additionally, evidence from shooting sports such as pistol, rifle, and archery also showed clear neural efficiency by many studies (Martins et al., 2022). Corroborating the neural efficiency hypothesis in closed-skill endurance sports, Ludyga et al. (2016) found that highly fit cyclists exhibit an increased alpha/beta ratio during exercise, indicating a more efficient cortical state characterized by the inhibition of task-irrelevant cognitive processes.

Evidence from precision sports further reinforces the neural efficiency model; for instance, Lizama et al. (2025) demonstrated that competitive golfers exhibit reduced cortical overload—evidenced by decreased absolute beta power and enhanced voluntary cortical inhibition—suggesting that expertise in closed-skill disciplines relies on minimizing neural energy expenditure to optimize performance. Furthermore, research on professional golfers (Yu et al., 2024) reveals that higher self-efficacy is associated with reduced frontal midline theta activity and superior putting accuracy, suggesting that in closed-skill sports, neural efficiency is achieved through the reduction of unnecessary top-down attentional interference during automated motor execution.

The neural efficiency model is also supported by resting-state network (RSN) analysis in world-class gymnasts. For instance, Huang et al. (2018) utilized independent component analysis (ICA) to demonstrate that elite gymnasts exhibit significantly decreased intra- and inter-network functional connectivity within the basal ganglia, anterior default mode networks, and left and right fronto-parietal networks. This global reduction in resting-state communication may reflect a more streamlined resting-state architecture where long-term motor skill automation leads to reduced metabolic and informational demands at rest. In addition, the reduction in dynamic network flexibility observed in elite gymnasts in another study can be characterized as a hallmark of neural efficiency (Cao et al., 2025).

### 3.2 Opposite evidence from open-skill sports

In open-skill sports, increases in brain volume and resting-state functional connectivity were observed in areas associated with executive control, motor sensation, vision, balance, and coordination (Yu et al., 2025). Converging evidence from soccer anticipation paradigms indicates that expertise under deceptive and temporally occluded conditions is associated with greater recruitment of executive, oculomotor, and action observation networks. In contrast with exhibiting global hypoactivation, high-skilled players demonstrate amplified engagement of task-relevant cortical and subcortical regions when perceptual uncertainty increases. Such findings are more consistent with a neural optimization framework emphasizing adaptive and context-sensitive neural amplification in open-skill environments (Bishop et al., 2013; Wright et al., 2013). Therefore, increased cortical excitability and a heightened neural responsiveness pattern was found among soccer players previously. Thus, Iwadate et al. (2005) demonstrated that soccer players exhibit the larger amplitudes of somatosensory event-related potentials (ERPs) during upper- and lower-limb tasks compared to non-athletes, indicating the plastic changes in somatosensory processing. Murakami et al. (2008) revealed a larger amplitude in soccer players compared to non-athletes, as well as the number of negative peaks of high-frequency oscillations (HFOs) from the posterior tibial nerve.

Recent high-field neuroimaging evidence also extends beyond classical hypoactivation accounts. For example, using 7-Tesla fMRI, Li et al. (2023) demonstrated that elite table tennis players exhibit increased dynamic resting-state activity (dALFF) in somatosensory and visual regions compared to non-athletes. This suggests that expertise in open-skill sports is associated with enhanced neural flexibility and “readiness” of functional networks, rather than a mere reduction in metabolic or electrical output. Moreover, another table tennis study revealed that P3 amplitude increased with the rise of expertise (Peng et al., 2022). Similar patterns of heightened neural engagement in response to environmental uncertainty have been observed in other open-skill domains. Neuroimaging evidence from various open-skill disciplines indicates that expertise may be associated with enhanced neural engagement rather than uniform hypoactivation. Thus, in a study of elite hockey players, experts demonstrated distinct patterns of cortical involvement in visuospatial decision-making compared to novices, including enhanced engagement of fronto-parietal networks supporting integrative processing (Wimshurst et al., 2016). Furthermore, a randomized controlled trial investigating action decision-making in basketball found that expert athletes demonstrated significantly greater activation within the inferior frontal gyrus and inferior parietal lobe compared to novices (Wu et al., 2013). These findings, coupled with more stable gaze patterns and higher accuracy, suggest that elite performance in open-skill sports relies on the strategic recruitment of attentional and sensorimotor networks rather than global neural economy. In addition, results from martial arts expertise (Sanchez-Lopez et al., 2014) further clarify the task-dependent nature of neural signatures. Research comparing attention-driven tasks reveals that while skilled athletes exhibit resource economy during cued, predictable responses, they demonstrate significantly greater prefrontal activity during sustained attention tasks that require simultaneous cognitive and motor control. This suggests that motor expertise does not result in a permanent state of hypoactivation, but rather enhances neural flexibility.

Evidence from badminton anticipation paradigms converges on a similar pattern of network-level specialization. Whole-brain fMRI studies demonstrate that expert players exhibit stronger activation within task-sensitive cortical regions during shuttle prediction, whereas novices show less focused or extra-network recruitment. Experts display enhanced functional connectivity between the medial frontal cortex and other regions implicated in action processing, including the posterior cingulate cortex, inferior parietal lobule, insula, and fusiform gyrus. Rather than reflecting reduced cortical expenditure, these findings indicate refined and integrated anticipation networks in skilled performers. Such patterns are consistent with neural optimization characterized by selective amplification and strengthened interregional coupling in response to complex, temporally constrained perceptual demands (Wright et al., 2011).

In line with findings in baseball (Nakamoto and Mori, 2008), experts in open-skill sports exhibit increased attentional resource allocation—evidenced by larger P300 amplitudes—rather than the reduced neural activity predicted by the traditional neural efficiency hypothesis. From previous evidence, Di Russo et al. (2006) also demonstrated that elite fencers exhibit significantly stronger prefrontal cortex activity and enhanced attentional modulation during complex decision-making tasks, suggesting that open-skill expertise relies on the heightened mobilization of neural resources to manage stimulus discrimination and motor inhibition. These findings align with our framework, suggesting that expertise in soccer and other open-skill sports may reflect strategic neural optimization and heightened functional readiness of frontoparietal systems, rather than the simple metabolic reduction predicted by the classical NEH. Collectively, these patterns suggest that open-skill expertise may involve more adaptable and integrative neural recruitment—consistent with a neural optimization perspective—rather than generalized reductions in activation magnitude.

## 4 From neural efficiency to neural optimization

To address these inconsistencies, we propose that expertise in open-skill sports may be characterized by neural optimization rather than global neural efficiency. Neural optimization does not imply a uniform reduction in cortical activity; instead, it reflects an adaptive, context-sensitive allocation of neural resources.

Based on the converging evidence reviewed above, neural optimization can be operationalized along four interrelated dimensions:

- **Task-specific recruitment:** Selective activation of task-relevant cortical regions rather than generalized suppression.
- **Temporal precision:** Enhanced oscillatory coordination and synchronization across frequency bands (e.g., theta-gamma coupling).
- **Dynamic reconfiguration:** Flexible network reorganization in response to shifting situational demands.
- **Interference control:** Reduced noise from task-irrelevant networks combined with preserved responsiveness in critical circuits.

Intriguingly, the dual-pathway model of sport-specific neuroplasticity proposed in the present review (neural efficiency vs. neural optimization) may parallel the fundamental dual-network architecture of the human attentional system. In this framework, expertise in closed-skill sports may primarily rely on the refinement of dorsal top-down attentional mechanisms, consistent with classical interpretations of neural efficiency. In contrast, mastery in highly dynamic open-skill environments such as soccer may depend on the heightened functional readiness of stimulus-driven reorienting systems associated with the Ventral Attention Network (Dong et al., 2024). Within this perspective, neural optimization can be interpreted as the adaptive tuning of networks specialized for the rapid detection and processing of behaviorally relevant external events:

- Closed skill → neural efficiency → Dorsal Attention Network (DAN)
- Open skill → neural optimization → Ventral Attention Network (VAN)

Thus, we propose that the neural signature of athletic expertise is not universal but ecologically constrained: while closed-skill disciplines tend to favor neural efficiency through the refinement of top-down control systems, open-skill sports such as soccer may rely on neural optimization characterized by heightened readiness of stimulus-driven attentional networks (bottom-up). However, while we emphasize the divergence between these pathways, expertise likely also involves the fluid interaction between DAN and VAN, allowing elite athletes to maintain top-down goal focus while remaining acutely responsive to dynamic environmental shifts. Therefore, this mapping should be interpreted as a heuristic conceptual framework rather than a strict neurobiological dichotomy, particularly given the well-established interaction between dorsal and ventral attentional systems.

In sum, although the present review is anchored in evidence from soccer research, soccer represents a prototypical open-skill sport characterized by continuous perceptual uncertainty, opponent interaction, and rapid decision-making. Therefore, neural patterns observed in soccer may provide a useful model for understanding broader principles of neuroplastic adaptation in dynamic sport environments. Nevertheless, extending this framework to other open-skill disciplines requires careful empirical validation. In addition, consistent with our dual-pathway model, extreme cases of motor automation, such as that observed in elite players during simple ankle rotations (Naito and Hirose, 2014), demonstrate localized neural efficiency in primary motor regions. However, we argue that such “Neymar-phenomenon” reflects the automation of isolated motor sub-routines rather than the global neural signature of expertise during complex, open-skill competition. In dynamic environments, the focus shifts from primary motor economy to executive optimization within the VAN.

## 5 Conclusion

The present synthesis supports a context-dependent framework of sport-related neuroplasticity in which neural optimization emerges as the dominant signature of expertise in dynamic open-skill disciplines. While classical interpretations of the NEH emphasize reduced cortical activation, accumulating evidence from soccer and other interactive sports suggests a more complex reality. In highly unpredictable environments, expertise is not necessarily expressed as doing “less” neurally, but as doing “better” through selective amplification and finely tuned coordination of executive networks.

By synthesizing evidence from 16 distinct athletic disciplines—equally representing closed-skill (e.g., golf, shooting, gymnastics, swimming) and open-skill (e.g., soccer, fencing, baseball, and basketball) domains—this review provides a preliminary conceptual framework supported by heterogeneous empirical evidence for a dual-pathway model. We propose that while closed-skill sports may favor metabolic economy, open-skill performance requires functional readiness and adaptive resource allocation. This perspective does not replace the NEH but reframes it within a broader ecological framework where the neural signature of mastery is fundamentally shaped by environmental uncertainty. Future investigations directly contrasting these disciplines under ecologically valid conditions will be critical for empirically testing this account and clarifying how sport-specific demands sculpt the expert brain.

Hemodynamic techniques such as fMRI and fNIRS have substantially advanced our understanding of spatially distributed activation patterns in sport-related tasks. The present framework emphasizes the potential complementarity of multimodal approaches. Open-skill performance unfolds across multiple temporal scales—from millisecond-level oscillatory coordination to slower network-level integration. Accordingly, hybrid designs combining electrophysiological and hemodynamic measures (e.g., EEG-fMRI or EEG-fNIRS) may offer a particularly informative avenue for future sport neuroscience research by simultaneously capturing spatial localization and fast neural synchronization dynamics. Such multimodal integration would allow more precise empirical testing of the neural optimization hypothesis proposed here.
